# Degradation of Natural *Undaria pinnatifida* into Unsaturated Guluronic Acid Oligosaccharides by a Single Alginate Lyase

**DOI:** 10.3390/md22100453

**Published:** 2024-10-02

**Authors:** Hui Wang, Jiaqi Wen, Nuraliya Ablimit, Kun Deng, Wenzhuo Wang, Wei Jiang

**Affiliations:** State Key Laboratory of Animal Biotech Breeding, College of Biological Sciences, China Agricultural University, Yuan Ming Yuan West Road No. 2, Haidian District, Beijing 100193, China; wh15666529202@163.com (H.W.); wjq18810755707@163.com (J.W.); aliya0814@163.com (N.A.); dengkun1103@163.com (K.D.); sz20213020199@cau.edu.cn (W.W.)

**Keywords:** alginate lyase, action mode, alginate oligosaccharide, catalytic mechanism, *Undaria pinnatifida*

## Abstract

Here, we report on a bifunctional alginate lyase (Vnalg7) expressed in *Pichia pastoris*, which can degrade natural *Undaria pinnatifida* into unsaturated guluronic acid di- and trisaccharide without pretreatment. The enzyme activity of Vnalg7 (3620.00 U/mL-culture) was 15.81-fold higher than that of the original *alg* (228.90 U/mL-culture), following engineering modification. The degradation rate reached 52.75%, and reducing sugar reached 30.30 mg/mL after combining Vnalg7 (200.00 U/mL-culture) and 14% (*w*/*v*) *U. pinnatifida* for 6 h. Analysis of the action mode indicated that Vnalg7 could degrade many substrates to produce a variety of unsaturated alginate oligosaccharides (AOSs), and the minimal substrate was tetrasaccharide. Site-directed mutagenesis showed that Glu^238^, Glu^241^, Glu^312^, Arg^236^, His^307^, Lys^414^, and Tyr^418^ are essential catalytic sites, while Glu^334^, Glu^344^, and Asp^311^ play auxiliary roles. Mechanism analysis revealed the enzymatic degradation pattern of Vnalg7, which mainly recognizes and attacks the third glycosidic linkage from the reducing end of oligosaccharide substrate. Our findings provide a novel alginate lyase tool and a sustainable and commercial production strategy for value-added biomolecules using seaweeds.

## 1. Introduction

Macroalgae (seaweed) account for about 90% of marine flora and around 50% of global photosynthesis among the wide variety of marine life [1]. Brown algae, one of the three major types of seaweed, is an integral component of marine ecosystems and a major marine biomass resource [2,3]. *Undaria pinnatifida* (Wakame) is an edible brown algae rich in polysaccharides with antioxidant, antibacterial and other biological properties activities, while the utilization efficiency still remains low [4].

Alginate is the predominant polysaccharide in brown algae, comprising ~17% to 47% of the dry weight, and the polysaccharide content of *U. pinnatifida* sporophylls can reach up to 52.52% [5,6]. Alginate is composed of two hexuronic acid residue, α-L-guluronic acid (G) and its C-5 epimer β-D-mannuronic acid (M), connected by 1,4-glycosidic linkages and arranged in various sequences that give rise to three distinct blocks: guluronate oligosaccharides (polyG), mannuronic oligosaccharides (polyM), and heteropolymer polyMG [7]. The major factors restricting the potential applications of alginate are its macromolecular structure, high viscosity, and low bioavailability [8]. Alginate oligosaccharides (AOSs) consisting of 2–25 monosaccharides can be produced by the physical, chemical, or enzymatic degradation of alginate [9]. AOSs are widely applied in the food, pharmaceutical, and biotechnology fields because of their water solubility and beneficial biological activities (immunomodulatory [10], antitumor [11], antibacterial [12], antioxidant [13], anti-inflammatory [14,15], and growth-promoting [16]). Enzymatic degradation of alginate or *U. pinnatifida* for AOS production is generally preferred over physical or chemical processes due to its low environmental impact, safety, site-specific cleavage reaction, high efficiency, and small amount of by-products [17]. Unsaturated AOSs produced by enzymatic degradation typically have functional groups with a conjugated alkene acid structure at the non-reducing end which display antioxidant activity stronger than that of saturated AOSs produced by chemical processes [18].

Alginate lyases (ALGs), a group of enzymes that specifically catalyze alginate degradation, have been isolated from marine algae, marine mollusks, and numerous marine and terrestrial bacteria, fungi, and viruses [19]. ALGs cleave O-C4 glycosidic bonds to uronic acid residues of alginate through β-elimination reaction, thereby generating a 4,5-unsaturated hexuronic acid residue at the non-reducing terminus of products [20]. In the Carbohydrate-Active enZymes (CAZy) database (cazy.org, accessed on 25 August 2024), ALGs are classified into multiple polysaccharide lyase (PL) families (PL5, -6, -7, -14, -15, -17, -18, -31, -32, -34, -36, and -39) [21]. More generally, ALGs can be assigned into two broad categories—exolytic and endolytic lyases—based on their action mode. Exolytic ALGs degrade oligosaccharides into unsaturated monomers, whereas endolytic ALGs cleave glycosidic linkages of alginate to yield unsaturated oligosaccharides (di-, tri-, and tetra-saccharides, termed delta or △2, △3, or △4) as major products [22]. The endolytic ALG from *Vibrio Splendidus* OU02 specifically breaks down alginate into mono-distributed trisaccharides [23]. In terms of substrate preference, ALGs can be categorized as polyG-specific (EC4.2.2.11), polyM-specific (EC4.2.2.3), and bifunctional (EC4.2.2.-) lyases [19]. The former two groups react preferentially with GG and MM blocks, whereas bifunctional lyases react with both polyG, polyM, and polyMG blocks and thereby degrade alginate more efficiently [24].

Technological advances in genetic engineering during the past decade have facilitated large-scale production of many ALGs. The recently developed *Pichia pastoris* expression system for ALGs has several advantages over the previously used original strain and *E. coli* expression systems: secretory expression, eukaryotic proper protein folding, high protein synthesis, and more suitability for industrial application [25]. Heterologous protein expression in *P. pastoris* can be enhanced by techniques such as cultural condition improvement, codon optimization, and native propeptide recruitment [26,27]. C. Zhu et al. engineered the recombinant ALG (Algt1) in *P. pastoris* to improve the potential of Algt1 for AOS preparation in high-concentration substrate [28].

There are few reports on the use of multi-enzymes or strain-enzymes to collaboratively degrade pretreatment brown seaweed to produce AOSs [29,30]. However, using a single efficient enzyme to directly degrade *U. pinnatifida* and convert it into value-added products is still an obstacle now. ALGs suitable for industrial applications are still in short supply. *Vibrio*, which are widely present in marine environments, have the capability to produce a range of highly efficient and stable ALGs [19]. We describe here the characterization of a novel PL7 family ALG (termed Vnalg7) from *Vibrio* sp. NJU-03 and evaluate the ability of the single enzyme (Vnalg7) to degrade *U. pinnatifida*. We also identify the catalytic sites and mechanism of Vnalg7 by site-directed mutagenesis.

## 2. Results and Discussion

### 2.1. Sequence Analysis of Vnalg7 and Enhanced Expression in P. pastoris

Information in the NCBI and CAZy databases indicated that gene *alg* (gene No. KY062661) in *Vibrio* sp. NJU-03 encodes an ALG (which we termed Vnalg7) belonging to family PL7. *alg* has a length of 1344 bp and encodes a 447 amino acid protein. The 22 N-terminal amino acids were predicted as a signal peptide sequence by SignalP 6.0 server program (http://services.healthtech.dtu.dk/service.php?SignalP, accessed on 25 August 2024). Analysis by ProtParam tool of ExPASY program (http://web.expasy.org, accessed on 25 August 2024) gave the predicted pI value 4.15 and theoretical molecular mass 48.11 kDa for Vnalg7. The *alg* sequence was optimized by upgrading the codon adaptation index (CAI) from 0.65 to 0.93 relative to the native sequence. Sequence alignment analyses revealed the following similarity values of Vnalg7 with PL7-family ALGs from various Gammaproteobacteria: *Vibrio cyclitrophicus* (MEZ9360292.1) 80.76%, *Vibrio breoganii* (WP_102430945.1) 63.75%, *Vibrio ishigakensis* (GAM76022.1) 60.78%, and *Vibrio ishigakensis* (GAM65289.1) 59.88%.

**Figure 1:**
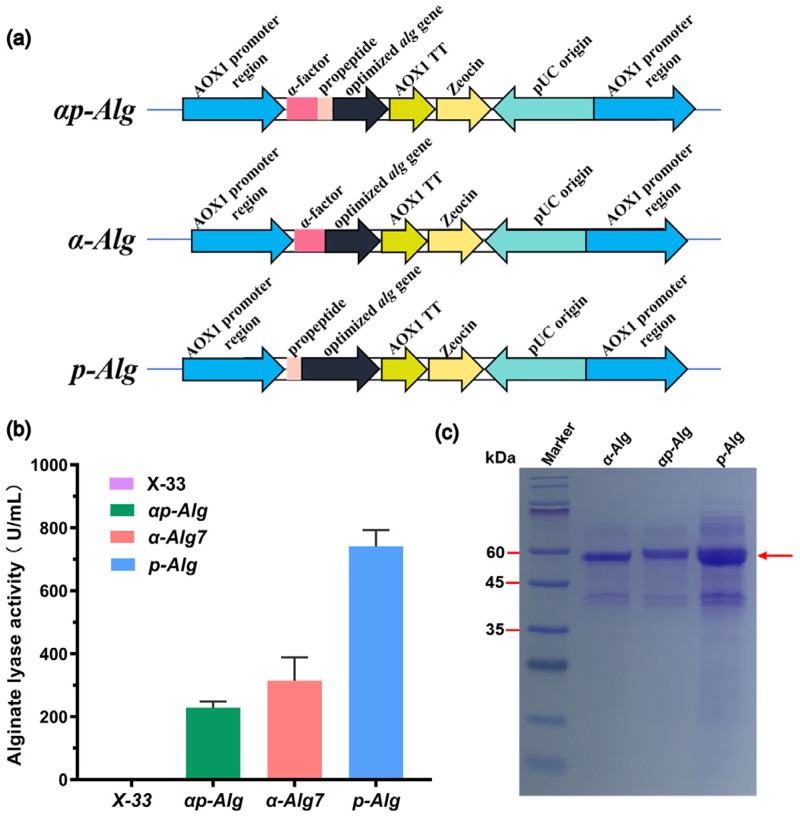
Enhancement of Vnalg7 expression in *P. pastoris* using propeptides. (**a**) Schematic representation of engineered recombinant Vnalg7 strains: *αp-Alg* (optimized *alg* gene with α-factor signal and propeptide), *α-Alg* (optimized *alg* gene with α-factor signal peptide), and *p-Alg* (optimized *alg* gene with propeptide). (**b**) Comparative extracellular Vnalg7 activity, in flask culture, toward alginate of above strains and X-33 (control). (**c**) SDS-PAGE analysis of above strains. Marker: ExcelBand Enhanced 3-color Regular Range Protein marker PM2510 (SMOBIO).

*P. pastoris* is a commonly used, well-studied industrial host for heterologous protein synthesis. Efficient recombinant protein production by *P. pastoris* is achieved by multi-level optimization strategies that integrate factors such as codon bias, signal peptides, gene dosages, and culture conditions [31]. We investigated the effects of propeptide insertion and codon optimization on Vnalg7 enzyme activity. *P. pastoris* recombinant strains were constructed through the transformation of linearized engineered plasmids pPICZαp-*alg*, pPICZα-*alg*, and pPICZp-*alg* and, respectively, termed *αp-Alg, α-Alg*, and *p-Alg* (Figure 1a). Positive transformants were screened on Yeast extract peptone dextrose (YPD) agar plates with 100 μg/mL Zeocin and confirmed by PCR (Figure S1).

Enzyme activity levels were measured following 120 h buffered minimal methanol medium (BMMY) incubation with substrate low-viscosity sodium alginate (LV-Algin) (blank control: Strain X-33). Activity of α-ALG was 313.83 U/mL, 1.40-fold higher than that of αp-ALG (228.90 U/mL-culture) (Figure 1b). Extracellular activity of p-ALG was 741.12 U/mL-culture, 3.20-fold higher than that of αp-ALG (Figure 1b). Target proteins of the three recombinant strains migrated with similar apparent Mw ~60 kDa (Figure 1c). Variation in the activities of the strains was presumably due to codon optimization and differences in protein concentrations resulting from changes in the signal peptide. The α-factor signal peptide of *P. pastoris* itself may be less efficient in directing heterologous protein secretion than some heterologous protein propeptides. Similarly, we previously observed 68.40% activity increase of an endoxylanase expressed in *P. pastoris* following the optimization and fusion of propeptide codons [32].

### 2.2. Bioreactor High-Density Fermentation of Vnalg7

**Figure 2:**
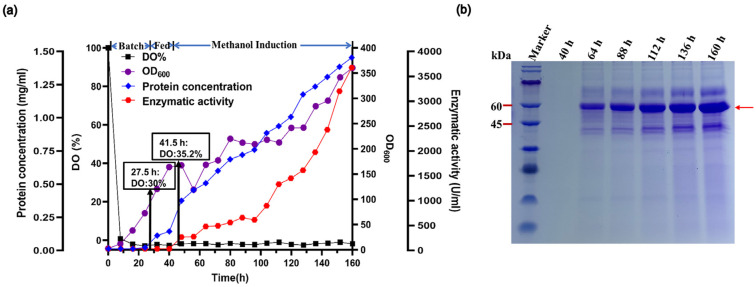
High-density fermentation of *p-Alg* in 7.5-L fermentor. (**a**) Time course profiles. Black square, DO%. Purple circle, cell density (OD_600_). Blue diamond, extracellular protein concentration. Red hexagon, extracellular Vnalg7 activity. (**b**) SDS-PAGE analysis of Vnalg7 extracellular protein at various times after induction. Marker: protein Mw marker (9–180 kDa).

Fed-batch fermentation is a commonly used, effective operating technique for increasing enzyme production. The high-cell-density fermentation technique, focused on the optimization of fermentation parameters, is often effective for the large-scale synthesis of recombinant proteins with enhanced enzyme activity and reduced costs [33]. We performed fed-batch fermentation of the engineered recombinant Vnalg7 strain *p-Alg* in a 7.5 L fermentor. Glycerol was the sole carbon source for 42 h following *p-Alg* inoculation. Dissolved oxygen (DO) levels increased rapidly as glycerol was consumed. After 42 h, Vnalg7 expression was induced by the addition of methanol. OD_600_ and enzyme activity were measured for samples collected at 8 h intervals. Agitation was maintained at 400–700 prm, and ventilation was 4–13 L/min throughout the fermentation cycle (Figure S2). Following 160 h fermentation growth, OD_600_ reached 360.50 (Figure 2a). The crude enzyme activity of the fermentation culture supernatant was 3620.00 U/mL-culture, 4.88-fold higher than that of the shake-flask culture (741.12 U/mL-culture) (Figure 2a) and much higher than values reported for ALGs scaled up by fed-batch fermentation of various *Vibrio*, including *Vibrio* sp. QY102 (52.8 U/mL) [34] and *Vibrio fortis* (560 U/mL) [30]. The protein concentration of the culture supernatant was 1.43 g/L (Figure 2a), the highest yield recorded to date for the *P. pastoris* recombinant expression of any ALG. Protein bands separated by SDS-PAGE were similar to those from the shake-flask culture (Figure 2b).

### 2.3. Enzymatic Characteristics of Recombinant Vnalg7

Enzymatic characteristics of p-ALG Vnalg7 were investigated using the substrate LV-Algin. Vnalg7 activity was maximal at pH 7.0 (Figure S3a) and the temperature 35 °C (Figure S3b). Previous studies of ALGs have generally shown optimal temperatures to be in the range 30–50 °C and optimal pH in the range of 7.0–9.0. Maximal activity of AlgA (8306.71 U/mg) was observed at 40 °C/ pH 7.5 [35], and that of PL7-family Algb from *Vibrio* sp. W13 at 30 °C/pH 8.0 [36].

Vnalg7 showed broad pH stability (>60%) in the range 7.0–11.0 following 1 h incubation at 35 °C (Figure S3c). Similar broad pH stability has been observed for other ALGs, e.g., A9mT from *Vibrio* sp. A9m [37]. Thermostability was maximal for 6 h preincubation at 35 °C, but much lower for preincubation at 45 °C or 55 °C (Figure S3d). Previous studies also reported an optimal temperature of 35 °C and a sharp decrease in activity at 60 °C for ALG AlgB from *Vibrio* sp. Ni1 [38]. The above findings indicate that Vnalg7 is an alkaline-stable ALG potentially useful for reactions under stressful conditions.

The effects of the metal ions and chemical reagents listed are summarized in Table S2. Vnalg7 activity was essentially unaffected by Na^+^ treatment (100.13%) but greatly reduced by Zn^2+^ (46.64%), Ni^2+^ (44.87%), and Cu^2+^ (26.29%). Activity was increased by Tween-20 (113.66%) but inhibited by EDTA (78.88%) and SDS (21.36%).

Kinetic parameters of Vnalg7 were evaluated using middle-viscosity sodium alginate (MV-Algin), LV-Algin, polyG, and polyM as substrates and determined at substrate concentrations ranging from 1 to 20 mg/mL. *K*_m_ (substrate concentration at which enzyme displays 50% of *V*_max_) and *V*_max_ (maximal catalytic velocity of enzyme) values were, respectively, 4.60 mg/mL and 2000.00 U/mg for MV-Algin, 5.00 mg/mL and 1666.67 U/mg for LV-Algin, 5.47 mg/mL and 1833.34 U/mg for polyG, and 7.42 mg/mL and 1166.67 U/mg for polyM (Table S3). *K*_m_ values were lower (i.e., substrate affinity higher) for alginate and polyG than for polyM, also indicating that Vnalg7 is a polyG-preferring bifunctional ALG. B. Zhu’s group reported that polyG- and polyMG-specific ALGs contain QIH in the conserved region, which is associated with substrate specificities of PL7-family ALGs [39]. The protein sequence alignment of Vnalg7 to PL7-family ALGs revealed the conserved region of QIH (Figure S4). Enzymatic characteristics of Vnalg7 are similar to those of two other PL7-family ALGs: AlyH1 from *Vibrio furnissii* H1 [40] and Alyw208 from *Vibrio* sp. W2 [41].

### 2.4. Action Mode of Vnalg7

**Figure 3:**
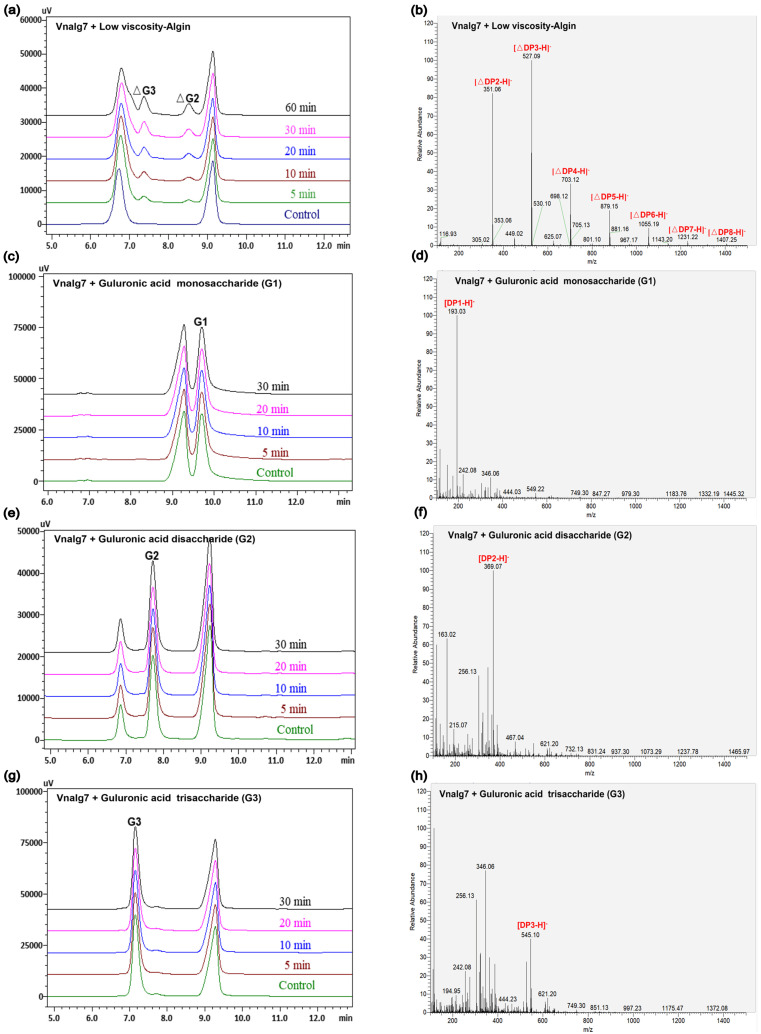

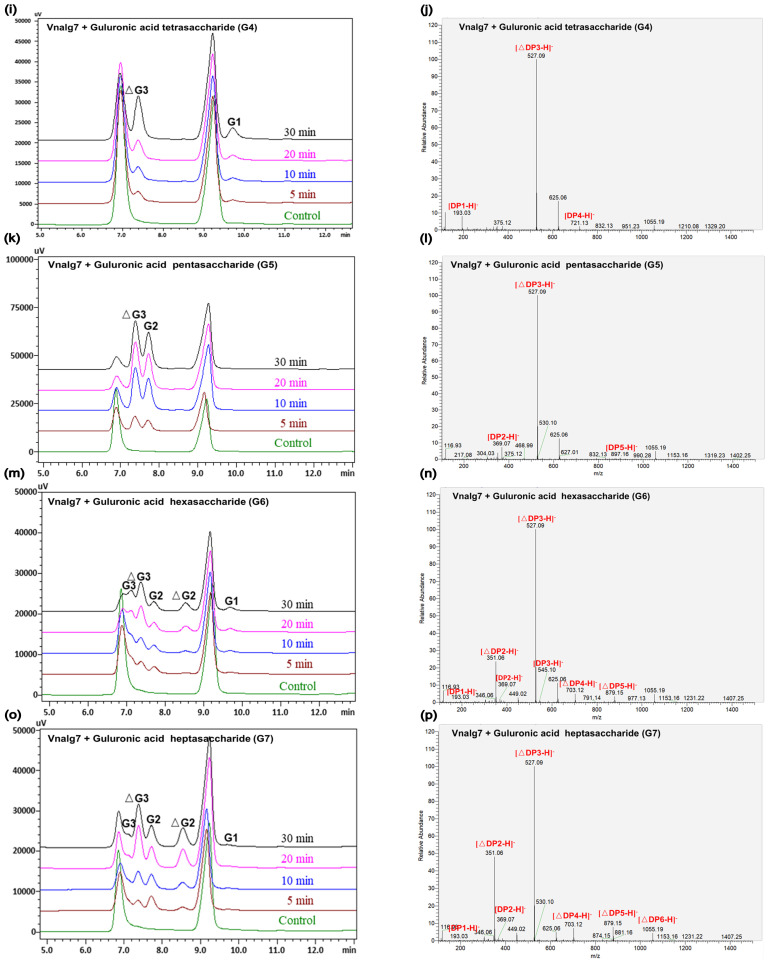
HPLC and LC-MS analysis of Vnalg7-catalyzed products of LV-Algin and guluronate oligosaccharides. (**a**,**b**) Composition analysis of Vnalg7-catalyzed products of LV-Algin. (**c**–**p**) HPLC and LC-MS analyses of Vnalg7-catalyzed products of guluronate oligosaccharides G1 to G7. Control: reaction system with inactivated Vnalg7 enzyme solution.

Vnalg7 enzymatic action mode was investigated using LV-Algin and standard saturated α-L-guluronic acid oligosaccharides as well as β-D-mannuronic acid oligosaccharides (G1 to G7, M1 to M7) as substrates. Results of the LC-MS analysis of standards are shown in Figure S5. HPLC in combination with LC-MS was used for the determination of product composition, thereby compensating for the low sensitivity of HPLC for the separation of highly polymerized AOSs. This analysis revealed that LV-Algin was degraded into various AOSs, while the major product components at longer reaction times were unsaturated disaccharides and trisaccharides (Figure 3a,b). Vnalg7 catalyzed the degradation of substrate endoglycosidic bonds and had excellent endolytic activity. It degraded guluronate and mannuronate oligomers but had less effect on M-enriched oligosaccharides (M1 to M7) (Figure S6). We therefore focused on the analysis of products of G-enriched oligosaccharides (G1 to G7). In regard to saturated guluronate oligomers, Vnalg7 did not degrade guluronic acid monosaccharide (G1), guluronic acid disaccharide (G2), or guluronic acid trisaccharide (G3) but degraded larger-size-defined fractions consistently with degraded saturated mannuronate oligomers (Figure 3c–h; Figure S6a–c). Guluronic acid tetrasaccharide (G4) was degraded into G1 and unsaturated guluronic acid trisaccharide (△G3) through Vnalg7 enzymatic action, indicating that G4 is the minimum identifiable substrate (Figure 3i,j). Among guluronic acid pentasaccharide (G5) products, the major components were △G3 and G2 (Figure 3k,l). Degradation products of guluronic acid hexasaccharide (G6) and guluronic acid heptasaccharide (G7) were more abundant than oligosaccharides of a lower polymerization degree (Figure 3m–p). However, the major products of G6 and G7 were △G3 and △G2, indicating that Vnalg7 acts in an endolytic manner but does not act on oligosaccharides with polymerization (DP) ≤ 3.

Degradation patterns differ among the various ALGs [42]. Most PL7-family ALGs studied to date are endolytic, the exceptions being two exolyases: VxAly7D from *Vibrio xiamenensis* QY104 [43] and VwAlg7A from *Vibrio* sp. W13 [44]. Vnalg7 efficiently degrades alginate in an endolytic manner to produce oligomeric AOSs, which provides a basis for potential applications in the food and pharmaceutical industries, particularly the production of value-added bioactive molecules (AOSs) [9].

### 2.5. Degradation of U. pinnatifida by Vnalg7 and Production of AOSs

**Figure 4:**
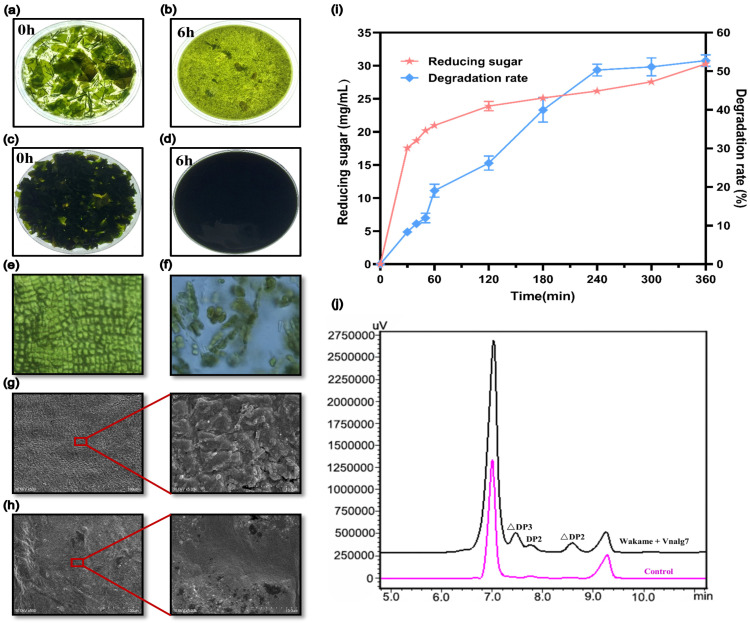
Enzymatic degradation structure changes and product analysis of *U. pinnatifida*. Visual observation of morphological changes of 2% (*w*/*v*) *U. pinnatifida* (**a**,**b**) and 14% (*w*/*v*) *U. pinnatifida* (**c**,**d**) degraded by Vnalg7. Optical microscopy images of the morphology of *U. pinnatifida* degraded by Vnalg7 for 0 (**e**) and 6 h (**f**), respectively. SEM images of undegraded (**g**) and degraded *U. pinnatifida* (**h**) for 6 h. (**i**) The 14% (*w*/*v*) *U. pinnatifida* degradation rate and reducing sugar released during 6 h enzymatic degradation process. (**j**) HPLC analysis of *U. pinnatifida* degradation products catalyzed by Vnalg7. Control as in Figure 3.

We evaluated the degradation efficiency of Vnalg7 toward the natural brown algae, *U. pinnatifida*, due to its high enzymatic activity on LV-Algin and MV-Algin. Vnalg7 displayed a strong degradation ability on *U. pinnatifida* raw materials and converted them into debris and soluble substances within 6 h (Figure 4a–d). Yu et al. reported that a combination of ALG and cellulase could efficiently hydrolyze the seaweed within 24 h [29]. The structural changes of *U. pinnatifida* after being degraded by Vnalg7 were observed by optical microscopy (Figure 4e,f) and scanning electron microscopy (SEM) (Figure 4g,h). The cell structure of undegraded *U. pinnatifida* was tightly arranged and relatively uniform in size (Figure 4g). *U. pinnatifida* exhibited a loose structure, increased cell gaps, and unevenly distributed, dense pores on the surface after degradation by Vnalg7 (Figure 4h). The variation in structure mainly resulted from the destruction of chemical bonds during enzymatic degradation to expose more hydrophilic groups, such as hydroxyl and carboxyl groups, whose exposure is crucial for the surface properties and biocompatibility of the material [45].

With the increase of enzymatic reaction time, both the degradation rate and the amount of released reducing sugars increased and finally stabilized (Figure 4i). The 14% (*w*/*v*) *U. pinnatifida* degradation rate reached 52.75%, and the reducing sugar reached 30.30 mg/mL after 6 h. The reducing sugar yield of 12% (*w*/*v*) *U. pinnatifida* powder degraded by ALGs VfAly7 (identified from *Vibrio fortis*) was only 23.50 mg/mL in 12 h [28]. To further determine the oligosaccharides composition of *U. pinnatifida* enzymatic liquid, the end-products were analyzed by HPLC. HPLC analysis revealed the products were mainly AOSs with △DP2, △DP3, and part of DP2 (Figure 4j). Unsaturated AOSs prepared by the enzymatic method have a variety of physiological activities such as antioxidant, anti-tumor, and immune-regulatory and have potential application value [46]. These findings indicated that Vnalg7 can degrade *U. pinnatifida* to produce AOSs and has the best effect reported so far for a single ALG to degrade *U. pinnatifida* without pretreatment.

### 2.6. Construction of Mutants and Analysis of Essential Catalytic Sites of Vnalg7

**Figure 5:**
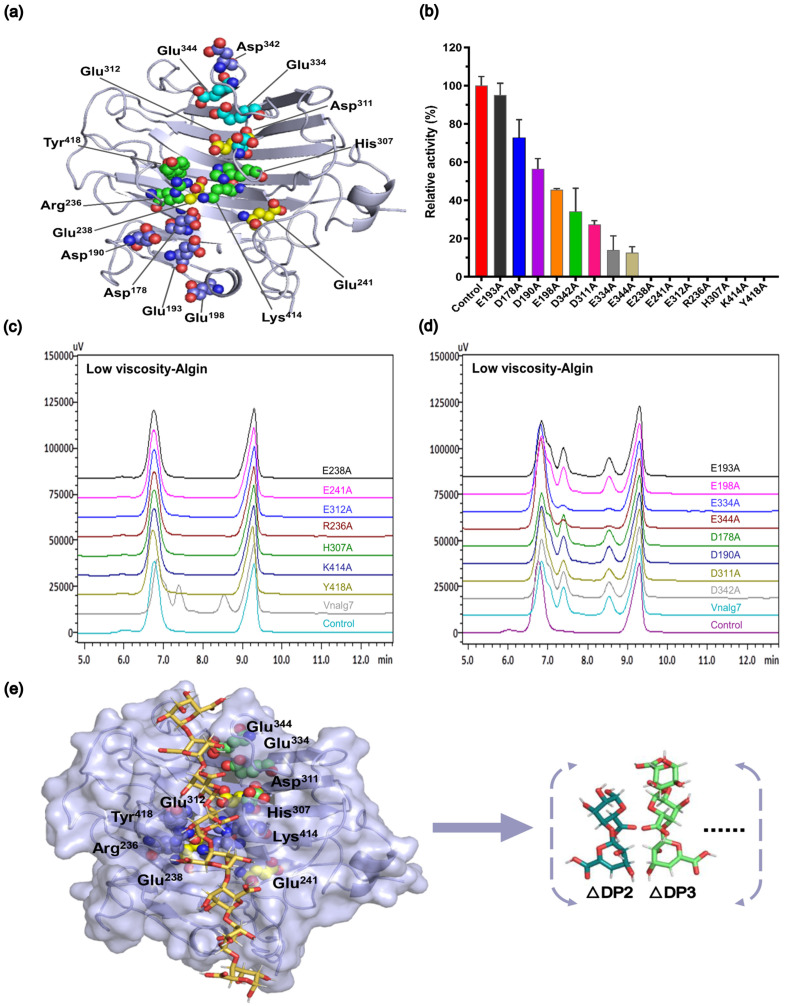
Construction of Vnalg7 mutants. (**a**) Protein 3D structure of Vnalg7 simulated and visualized using software programs AlphaFold2 and PYMOL (version 1.8.6). Predicted residues for mutants are labeled. (**b**) Enzyme activities of Vnalg7 (control) and constructed mutants to degrade LV-ALgin. (**c**,**d**) HPLC analysis of LV-Algin degradation products catalyzed by Vnalg7 and its mutants. Control as in Figure 3. (**e**) Molecular docking studies of Vnalg7 showing substrates and main products in predicted active sites during alginate degradation.

Since Vnalg7 has efficient enzymatic activity, we further explored its mechanism of degrading alginate. AlphaFold2 and SWISS-MODEL were utilized to predict the structure of Vnalg7, based on the results of multiple-sequence alignment. The root-mean-square deviation (RMSD) of the prediction models is 0.68 Å (Figure S7), indicating that the model predicted by homology modeling and AlphaFold2 is effective, ensuring the reliability of the prediction model. A preliminary determination of the active pocket of Vnalg7 was made considering the conserved site of PL7-family ALGs [47]. Amino acid residues that are highly conserved within the PL7 family may interact directly with the sugar residues occupying each subsite. Vnalg7 mutants were then designed and constructed using site-directed mutagenesis for the determination of essential catalytic sites of Vnalg7 involved in substrate recognition and catalytic activity (Figure 5a). On SDS-PAGE, recombinant proteins expressed by the mutants migrated as ~60 kDa bands, consistently with values for Vnalg7 (Figure S8a,b). The enzyme activity levels were similar to the Vnalg7 values for mutants E193A, D178A, D190A, E198A, and D342A; strongly reduced (by ~80–90%) for E334A, E344A, and D311A; and zero for E238A, E241A, E312A, R236A, H307A, K414A, and Y418A (Figure 5b). Enzymatic products of LV-Algin degraded by Vnalg7 or the mutants were identified by HPLC. Product composition analysis revealed that unsaturated disaccharides and trisaccharides, normally the major products, were produced by alginate degradation for E193A, D178A, D190A, E198A, and D342A but not for E238A, E241A, E312A, R236A, H307A, K414A, or Y418A (Figure 5c,d). The amounts of enzymatic products were significantly lower for E334A, E344A, and D311A than for Vnalg7 (Figure 5d).

These findings, taken together, indicate that amino acid’s Glu^238^, Glu^241^, Glu^312^, Arg^236^, His^307^, Lys^414^, and Tyr^418^ are the essential catalytic sites of Vnalg7 responsible for alginate catalysis activity, whereas Glu^334^, Glu^344^, and Asp^311^ may play accessory catalytic roles (Figure 5e). Arg^236^, His^307^, Lys^414^, and Tyr^418^ are the essential catalytic sites in the conserved region of PL7-family ALGs [22]. Detailed functions of essential and auxiliary catalytic sites in the degradation of guluronic acid oligosaccharides with varying DP values remain to be clarified.

### 2.7. Catalytic Mechanism of Vnalg7

The functions of essential and auxiliary catalytic sites were investigated using G7, M7, G6, M6, G5, M5, G4, and M4 as substrates to clarify the catalytic mechanism of Vnalg7. Vnalg7 and its mutants had no notable effect on mannuronic acid oligosaccharides (Figure S9e–h) and did not differ in active sites from guluronic acid oligosaccharides, and this topic was therefore not further pursued. In regard to guluronic acid oligosaccharides, E238A, E241A, E312A, R236A, H307A, K414A, and Y418A were shown by HPLC analysis to have no ability to degrade substrates G7, G6, G5, or G4 (Figure S9a–d), consistently with findings for alginate degradation. The amino acid’s Glu^238^, Glu^241^, Glu^312^, Arg^236^, His^307^, Lys^414^, and Tyr^418^ therefore appear to play active roles in catalytic reactions of G4 to G7 as essential catalytic sites. D311A, E334A, and E344A displayed some production during the degradation of G7, G6, and G5 but at reduced levels, whereas there was no production when G4 was used as the substrate (Figure S9a–d).

**Figure 6:**
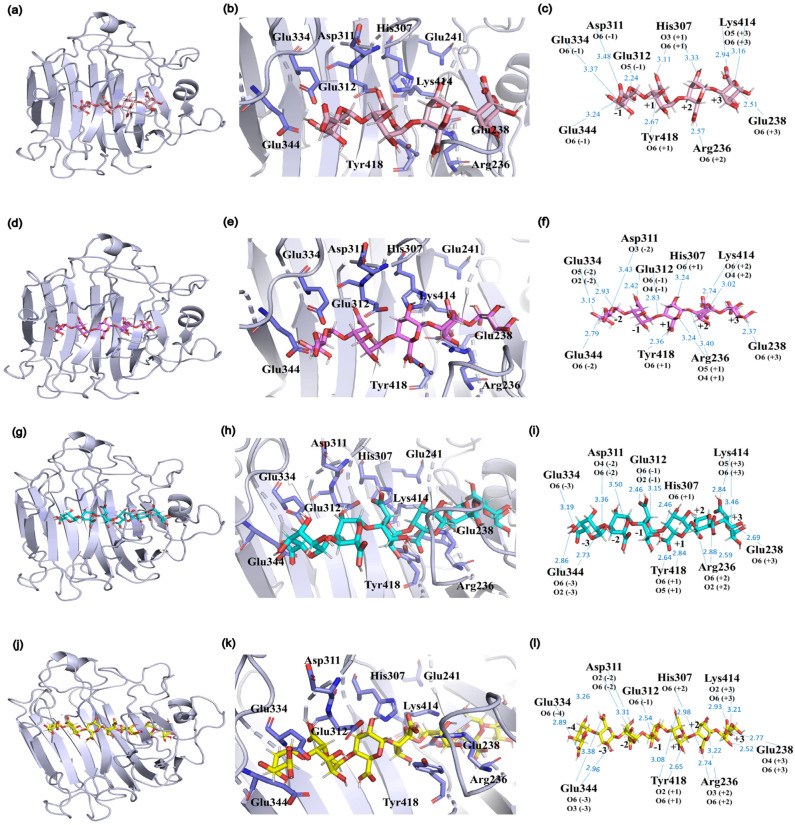
Substrate binding profiles of Vnalg7. Molecular docking studies revealed positions of substrates G4 (**a**), G5 (**d**), G6 (**g**), and G7 (**j**) in the predicted active pocket. Close-up views of the positional relationships between substrates G4 (**b**), G5 (**e**), G6 (**h**), and G7 (**k**) and the catalytic site amino acid residues in the active pocket. Schematic representation of the interactions formed between substrates G4 (**c**), G5 (**f**), G6 (**i**), and G7 (**l**) and the amino acid residues of the catalytic sites. Hydrogen bonds are indicated with blue dashed lines. The catalytic site amino acid residues are colored in purple, G4 in pink, G5 in rose red, G6 in cyan, and G7 in yellow.

**Figure 7:**
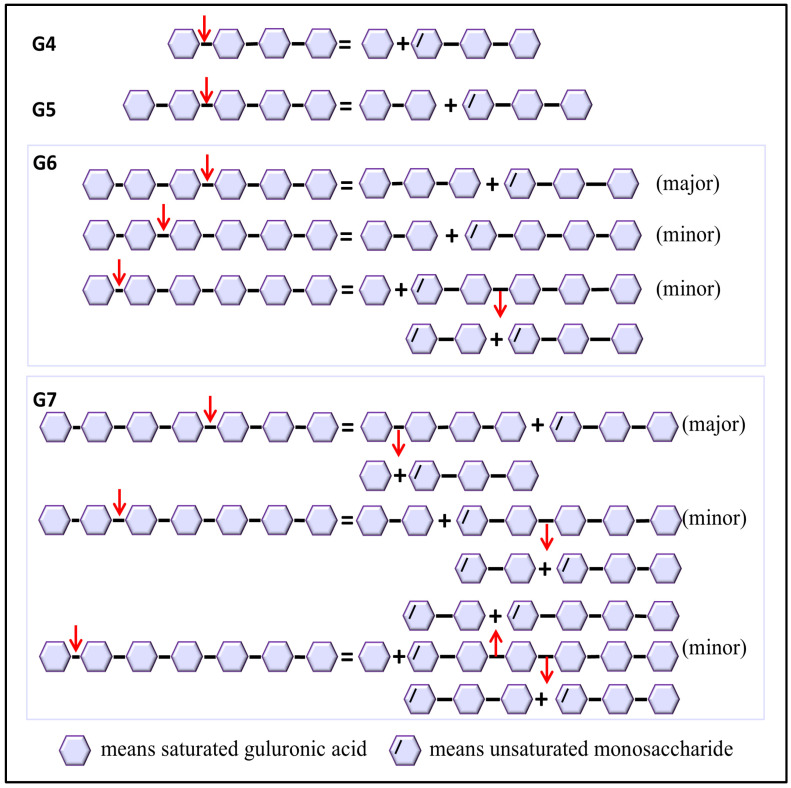
Catalytic mechanism of Vnalg7. Putative schematic model of catalytic mechanism of Vnalg7 toward various size-defined saturated G-enriched oligosaccharides. Arrows mean the position of linkage attacked by Vnalg7.

The degradation patterns and catalytic sites involved vary depending on the substrate. Molecular docking of various substrates into the Vnalg7 protein structure was examined to elucidate catalytic mechanisms. Three-dimensional structural simulations indicated that Vnalg7 catalytic sites were located on the surface of the active pocket in the shape of a cylindrical channel, with the exception of Glu^241^ (Figure S10a–c). The inner location of Glu^241^ suggests its possible involvement in electron and proton transfer processes during catalytic reactions [48]. Substrates G4 to G7 were docked in the catalytic region located between essential sites (Figure 6a,d,g,j). G4 was bound to the subsites of −1 to +3, generating G1 and △G3 (Figure 6a–c and Figure 7). G5 was degraded into G2 and △G3 bound through the −2 to +3 subsites (Figure 6d–f and Figure 7). When more highly polymerized oligosaccharides (G6, G7) were degraded, G6 was mainly bound to the subsites between −3 and +3, G7 was chiefly bound to the subsites of −4 to +3, and the longer glycosidic chains were processed by multiple catalytic sites to produce a variety of oligosaccharides, with △G3 as a major component (Figure 6g–l and Figure 7). Based on the product composition results, G6 and G7 also have other minor subsite binding modes (Figure 3m–p and Figure 7). Combined with the positions and interactions of the oligosaccharide residues with the catalytic amino acid sites within the active pocket and the results of protein multiple-sequence alignments, the conserved residues of Vnalg7-Arg^236^, Glu^238^, His^307^, Lys^414^, and Tyr^418^ (located in the three conserved regions QI(V)H, RXEL(V)R, and YFKXGXYXQ of the PL7-family ALGs) were found to be involved in forming interactions with the C-5 carboxyl groups at the +1 to +3 subsites of the oligosaccharides (Figure 6c,f,i,l and Figure S4). For G4, the residues Asp^311^, Glu^312^, Glu^334^, and Glu^344^ bound to its −1 subsite. For G5, G6, and G7, the residue Glu^312^ as the essential catalytic site chiefly formed hydrogen bonds with the C-5 carboxyl group of the −1 subsite, while Asp^311^, Glu^334^, and Glu^344^ mainly formed hydrogen bonds with hydroxyl or carboxyl groups of the other “-” subsites (Figure 6c,f,i,l). Q. Lyu et al. reported that amino acid residues in the conserved region of PL7-family ALGs were involved in substrate binding at the “+” section, while their “−” section might exhibit diverse substrate binding profiles [49]. These results indicated that the reducing end of oligosaccharides was preferentially located at the +3 subsite, and Vnalg7 mainly attacked the third glycosidic linkage (−1 to +1 subsites) from the reducing end of oligosaccharide substrates. The current study reported that most ALGs in the PL7 family mainly cleaved the third glycosidic linkage at the reducing end, with trisaccharide as the main product, through crystal structure and oligosaccharide degradation studies [50,51]. Information on the position and distance between amino acid residues was consistent with experimental results. Inter-residue spatial distances between Asp^311^, Glu^334^, Glu^344^, and essential catalytic sites are in the range 20.70–25.40 Å (Figure S10b,c), which provides sufficient space for the placement of G4 (17.00 Å), G5 (21.20 Å), G6 (23.00 Å), and G7 (25.00 Å). Considering the above results, we speculate that the Asp^311^, Glu^334^, and Glu^344^ mutations completely lost the degradation effect on G4, which might be due to the shorter length of G4 than the other three oligosaccharides, and the essential cleavage subsite −1 could not form a hydrogen bond interaction with the mutants D311A, E334A, and E344A.

## 3. Materials and Methods

### 3.1. Materials

*E. coli* (strain DH5α; used as cloning host), expression vector pPICZαA, and *P. pastoris* strain X-33 (used as protein expression host) are maintained in our laboratory. Plasmid extraction kits were from Tiangen Biotech (Beijing). PCR reagents and restriction endonucleases were from New England Biolabs (Ipswich, MA, USA). DNA markers were from Real-Times Biotechnology Co. (Beijing). Protein markers were from SMOBIO Technology, Inc. (Hsinchu, Taiwan). DNA polymerase and Mut Express II Fast Mutagenesis Kit V2 were from Vazyme Biotech Co. (Nanjing, China). MV-Algin (brown algae origin, viscosity ≥2000 mPa·s, 2% in H_2_O; CAS 9005-38-3, A2033) and LV-Algin (brown algae origin, viscosity 4–12 mPa·s, 1% in H_2_O; CAS 9005-38-3, A1112) substrates were from Sigma-Aldrich (St. Louis, MO, USA). PolyG (average Mw ∼5.5 kDa, M/G ratio 0.20, purity ∼95%), polyM (average Mw ∼6.44 kDa, M/G ratio 12.56, purity ∼80%), G1 to G7, and M1 to M7 (reported purity ≥95%) were from Qingdao BZ Oligo Biotech Co. (Qingdao, China). Standards were validated by HPLC and LC-ESI-MS results. Dried *U. pinnatifida* was from Dalian Yongtai Food Co. Other chemicals used were of analytical reagent grade and commercially available.

### 3.2. Construction of Recombinant Vectors and Expression of Vnalg7

Full-length ALG gene sequence (Gene ID KY062661; 1344 bp) from *Vibrio* sp. strain NJU-03 was optimized according to yeast codon usage bias (Java Codon Adaptation Tool; https://bio.tools/jcat, accessed on 25 August 2024) and sent to the company (Sangon Biotech, Shanghai, China) to synthesize and clone it into plasmid pPICZαA. ALG variants, plasmids pPICZα-*alg* (with α-factor signal peptide) and pPICZp-*alg* (with native propeptide), were amplified from designed plasmid pPICZαp-*alg* (with α-factor signal peptide and native propeptide) using corresponding PCR primers (Table S1) by one-step PCR [52]. Each recombinant vector was transformed into *E. coli* DH5α. Transformants were selected on LB plates with 50 μg/mL Zeocin (Invitrogen Corp.; Carlsbad, CA, USA), and confirmed by PCR and DNA sequencing. Recombinant plasmids were extracted, linearized using restriction endonuclease *Sac*I, concentrated, and electroporated into *P. pastoris* X-33 competent cells as described previously [53], generating engineered strains *αp-Alg, α-Alg*, and *p-Alg* (control strain: *P. pastoris* with pPICZαA empty vector). Positive transformants were selected on YPD agar plates with 100 μg/mL Zeocin. Integration of target gene into *P. pastoris* genome was confirmed by PCR using primer pair AOX-F/ AOX-R (Table S1). Recombinant colonies underwent protein expression induction in BMMY as described previously [54]. Enzyme supernatants were analyzed by 12% Tricine-SDS-PAGE. Protein bands were visualized by staining with Coomassie Brilliant Blue R-250 (Bio-Rad Laboratories; Carlsbad, CA, USA). Protein concentrations were determined by Bradford method with BSA as standard [55].

### 3.3. High-Density Fermentation Culture

Improved recombinant strain *p-Alg* (10% *v*/*v*) was inoculated into 5 L basal salt medium and fermented in a 7.5-L fermentor (Shanghai Boxing Bio-engineering Equipment Co.). Temperature 30 °C and pH 5.5 were maintained through monitored ammonium hydroxide (50% *v*/*v*) supplementation. Glycerol was the sole carbon source in batch phase. DO was adjusted to low-level with aeration rate 4–13 L/min and agitation 400–700 rpm. During fed-batch phase, as DO level increased rapidly, 200 mL 50% (*v*/*v*) glycerol with 1.2% (*v*/*v*) PTM1 *P*. *pastoris* trace element solution was added at 18 mL/h/L. During methanol feeding phase, after glycerol was exhausted, 0.2–0.5% (*v*/*v*) methanol mixed with 1.2% PTM1 was pumped into the fermentor through the bioreactor autocontrol system. Culture supernatant samples were taken at 8 h intervals, and cell concentration (OD_600_), protein concentration, and enzyme activity were measured.

### 3.4. Enzyme Activity Assay and Biochemical Characterization of Vnalg7

Vnalg7 enzyme activity was evaluated by UV absorption method [56], based on the formation of a double bond between C4 and C5 at the non-reducing terminus by β-elimination. A mixture of 0.1 mL appropriate enzyme dilution and 0.9 mL 1.0% (*w*/*v*) LV-Algin was incubated in 50 mM sodium phosphate buffer (pH 7.0) for 10 min at 35 °C, and the reaction was terminated by 10 min immersion in boiling water (control: same assay mixtures with deactivated enzyme). Enzyme activity was determined based on increased absorbance at wavelength 235 nm (A_235_), with one activity unit (U) defined as amount of enzyme required to increase A_235_ by 0.1 per minute and specific activity defined as units per mg protein.

Vnalg7 enzyme properties were evaluated using LV-Algin as substrate. Optimal pH values were determined in the range 2.0 to 11.0, using the buffers (each 50 mM) glycine-HCl (pH 2–3), sodium citrate (pH 3–4), sodium acetate (pH 4–6), sodium phosphate (pH 6–8), and Tris-HCl (pH 8–11). pH stability was evaluated by preincubating enzyme in buffers without substrate for 1 h at 4 °C, then measuring residual activity under standard assay conditions.

Optimal temperature for Vnalg7 activity was evaluated at each 5 °C interval within the range 20–65 °C, using 50 mM phosphate buffer (pH 7.0). Enzyme thermostability was determined by measuring residual activity under standard conditions after 6 h preincubation without substrate at 35, 45, and 55 °C.

Effects of various metal ions and chemical reagents on Vnalg7 activity were evaluated using 5 mM solutions of Ni^2+^, Co^2+^, Al^3+^, Na^+^, Mn^2+^, Zn^2+^, Ca^2+^, Cu^2+^, Mg^2+^, and K^+^ as well as 0.1% (m/v) SDS, 1 mM EDTA, and 0.05% (*v*/*v*) Tween-20 under standard reaction conditions (control: mixture without additive).

Enzyme kinetic assays were performed at substrate concentrations ranging from 0 to 20 mg/mL under optimal pH and temperature conditions. Kinetic parameters *V*_max_ and *K*_m_ were calculated by non-linear regression fit directly to the Michaelis–Menten kinetic equation. All assays were performed in triplicate.

### 3.5. Action Mode of Vnalg7

Vnalg7 action mode was evaluated based on analysis of degradation product components of 1% (*w*/*v*) LV-Algin and 5 mg/mL AOSs (G1 to G7, M1 to M7). Reaction mixtures were incubated in 50 mM sodium phosphate buffer (pH 7.0) at 35 °C for durations 0, 5, 10, 20, 30, and 60 min. Blank controls: substrates and inactive enzyme mixtures. Degradation products were analyzed by HPLC system (model LC-20A; Shimadzu; Kyoto, Japan) comprising CBM-20A controller and RID-10A refractive index detector, using ROA-organic acid H^+^ (8%) column (Phenomenex; Torrance, CA, USA) with column temperature 50 °C and mobile phase 5 mM H_2_SO_4_ (flow rate 0.6 mL/min). For more precise quantification of composition and Mw of degradation products, oligosaccharides were analyzed by LC-ESI-MS (Thermo Scientific, Waltham, MA, USA) using Waters ACQUITY UPLC HSS T3 column (2.1 × 100 mm, 1.8 μm) (negative ion mode; ion spray voltage 3.5 kV; source temperature of 250 °C). Mobile phase gradient elution involved mixtures of mobile phases A (0.1% formic acid) and B (100% acetonitrile) in various proportions (2 min, 98%A and 2%B; 7 min, 5%A and 95%B; 10 min, 98%A and 2%B). Standards: G1–G7 and M1–M7.

### 3.6. Degradation of U. pinnatifida by Vnalg7 and Composition Analysis of Products

Degradation of unpretreated 14% (*w*/*v*, alginate may account for ~17% to 47% of dry weight) *U. pinnatifida* was performed by mixing Vnalg7 (200.00 U/mL-culture) and an appropriate amount of 50 mM sodium phosphate buffer (pH 7.0) and incubating (temperature 35 °C) for 6 h with constant agitation (200 rpm). The reaction was stopped after heating at 100 °C for 10 min. The structures of degraded and natural (control) *U. pinnatifida* were observed and compared using optical microscopy (100×, DS-Fi1, Nikon, Japan) and SEM (SU8010 system, Hitachi, Japan). Before SEM analysis, the materials were covered with a gold layer (15 nm), and then images of different scales (500× and 5000×) were obtained.

Degradation products were collected at different times and centrifuged (13,400× *g*, 5 min). The supernatant was used to determine the released reducing sugar by the 3,5-dinitrosalicylic acid (DNS) method [57], while the precipitate was dried at 105 °C to estimate degradation rate using the following equation [58]:

*U. pinnatifida* degradation rate = (1 − W_T_/W_C_) × 100%

where W_T_ and W_C_ are the dry weights of the recovered *U. pinnatifida* sample after treatment with Vnalg7 and inactivated Vnalg7, respectively.

### 3.7. Construction of Vnalg7 Mutants

Arg^236^, His^307^, Lys^414^, and Tyr^418^ were considered as possible Vnalg7 catalytic sites based on Basic Local Alignment Search Tool (BLAST) analysis (http://blast.ncbi.nlm.nih.gov/Blast.cgi, accessed on 25 August 2024; National Center for Biotechnology Information [NCBI]) and protein multiple-sequence alignment (Figure S4). Multiple-sequence alignment was performed by Clustal Omega (http://www.clustal.org/omega, accessed on 25 August 2024), and the results were visualized using Jalview software (version 2.11.1.4). AlphaFold2 (https://alphafold2.biodesign.ac.cn/, accessed on 25 August 2024) and SWISS-MODEL server (http://swissmodel.expasy.org, accessed on 25 August 2024) were used for 3D structure simulation of the recombinant protein, with protein ALG AlyB (sequence similarity 57.69%; PDB ID. 5zu5.1) [59] as template. PyMOL Molecular Graphics System (http://pymol.org/2, accessed on 25 August 2024), DockingPie plugin, and Vina Docking program were used for structure visualization, site spatial location determination, and molecular docking [60]. Residues Glu^193^, Glu^198^, Glu^238^, Glu^241^, Glu^312^, Glu^334^, Glu^344^, Asp^178^, Asp^190^, Asp^311^, and Asp^342^ were identified as Vnalg7 active catalytic sites based on multiple-sequence alignment with AlyB protein sequence (Figure S4). Vnalg7 mutants were constructed by site-directed mutagenesis and designated R236A, H307A, K414A, Y418A, E193A, E198A, E238A, E241A, E312A, E334A, E344A, D178A, D190A, D311A, and D342A, corresponding to the above residues. Mutant genes were amplified from plasmid pPICZp-*alg* using corresponding primer pairs (Table S1). Transformation of mutant plasmids into *P. pastoris* and assays of protein expression, enzyme activity, and enzymatic properties were performed as described previously. Control: variant vector pPICZp-*alg* transformed into *P. pastoris*.

## 4. Conclusions

In conclusion, we characterized a PL7-family ALG (Vnalg7) originated from marine bacterium *Vibrio* sp. NJU-03 and evaluated its ability for the utilization of natural *U. pinnatifida* without pretreatment. Vnalg7 is a bifunctional enzyme that can degrade polyG and polyM, improving the efficiency of a single enzyme in degrading *U. pinnatifida* or alginate to value-added biomolecules (AOSs). Vnalg7 enzyme activity was enhanced by native propeptide recruitment and high-density optimized fermentation in *P. pastoris*. Site-directed mutagenesis studies revealed that residues Glu^238^, Glu^241^, Glu^312^, Arg^236^, His^307^, Lys^414^, and Tyr^418^ are the essential catalytic sites of Vnalg7, while Asp^311^, Glu^334^, and Glu^344^ are auxiliary sites in the degradation of most AOSs. Molecular docking of Vnalg7 and substrates clarified its catalytic mechanism for the degradation of alginate and common guluronic acid oligosaccharides. Future studies could focus on structural modifications to Vnalg7 to improve its stability and catalytic efficiency under industrial production conditions. Exploring the commercial potential of value-added biomolecules (AOSs) produced by degradation of natural brown algae using VnAlg7 in the agricultural, pharmaceutical, and food fields could help to promote the application of ALGs and AOSs in sustainable biotechnology and provide new perspectives for research in related fields.

## Data Availability

The original data presented in the study are included in the article/Supplementary Material; further inquiries can be directed to the corresponding author.

## Supplementary Materials

The following supporting information can be downloaded at: https://www.mdpi.com/article/10.3390/md22100453/s1. Figure S1. PCR confirmation of constructed engineered strains. Positive variants and *P. pastoris* X-33 (control strain, without target gene) were identified using the PCR primer pair AOX-F/AOX-R. Marker: 1-kb DNA markers. Positive: plasmids pPICZα-alg, pPICZp-alg, and pPICZαp-alg. Negative: X-33; Figure S2. Agitation and aeration variation in high-density fermentation of *p-Alg* in the 7.5-L fermenter. Black square, agitation. Purple circle, aeration. Figure S3. Enzymatic characteristics of Vnalg7 in engineered strain p-ALG. (a) Effects of pH on enzyme activity. (b) Effects of temperature on enzyme activity. (c) pH stability following 1 h preincubation in pH buffers shown in (a). (d) Thermostability in phosphate buffer, at three temperatures; Figure S4. Multiple-sequence alignment of Vnalg7 and PL7 family ALGs cited in this study. Residues Glu^193^, Glu^198^, Glu^238^, Glu^241^, Glu^312^, Glu^334^, Glu^344^, Asp^178^, Asp^190^, Asp^311^, Asp^342^, Arg^236^, His^307^, Lys^414^, and Tyr^418^ (order number in Vnalg7) which may participate in the catalytic process are highlighted in red. Three conserved regions in the PL7 family: QI(V)H, RXEL(V)R, and YFKXGXYXQ are boxed in black; Figure S5. LC-MS analysis of LV-Algin and guluronate oligosaccharide standards with various DP values; Figure S6. HPLC analysis of Vnalg7-catalyzed products of mannuronate oligosaccharides. Time course of Vnalg7-catalyzed hydrolysis of mannuronic acid oligosaccharides with various DP values. (a-g) mannuronic acid monosaccharide (M1), disaccharide (M2), trisaccharide (M3), tetrasaccharide (M4), pentasaccharide (M5), hexasaccharide (M6), and heptasaccharide (M7), respectively, as indicated in panel captions. Control: reaction system with inactivated Vnalg7 enzyme solution; Figure S7. Predictive 3D model of Vnalg7 by AlphaFold2 (purple) and SWISS-MODEL (cyan); Figure S8. SDS-PAGE analysis of Vnalg7 and its mutants; Figure S9. Enzymatic activity of Vnalg7 and its mutants. HPLC analysis of hydrolysates of substrates G7 (a), G6 (b), G5 (c), and G4 (d). (e–h) Hydrolysates of M7, M6, M5, and M4, respectively, as indicated in panel captions. Control as in Figure S6; Figure S10. Schematic representation of Vnalg7 active pocket positions and intercatalytic site distances. (a) Surface representation of 3D structure of Vnalg7, showing catalytic amino acid residues in predicted active pocket. (b, c) Schematic structural representations of catalytic region, with distances (Å) between catalytic residues indicated; Table S1. Oligonucleotide primers used in this study; Table S2. Effects of metal ions (5 mM solution) and chemical reagents on Vnalg7 activity; Table S3. Kinetic parameters *K*_m_ and *V*_max_ of Vnalg7 toward various substrates.

## Chemical Compounds Studied in This Article

Guluronic acid monosaccharide (PubChem CID: 446401); Guluronic acid disaccharide (PubChem CID: 93532748); Guluronic acid trisaccharide (PubChem CID: 91857798); Guluronic acid tetrasaccharide (PubChem CID: 91857005); Guluronic acid pentasaccharide (PubChem CID: 162642258); Guluronic acid hexose (PubChem CID: 156619317); Guluronic acid heptose (PubChem CID: 162642246); Mannuronic acid monosaccharide (PubChem CID: 446401); Mannuronic acid disaccharide (PubChem CID: 93532748); Mannuronic acid trisaccharide (PubChem CID: 91857798); Mannuronic acid tetrasaccharide (PubChem CID: 91857005); Mannuronic acid pentasaccharide (PubChem CID: 162642258); Mannuronic acid hexose (PubChem CID: 156619317); Mannuronic acid heptose (PubChem CID: 162642246).

## Abbreviations

Alginate lyases (ALGs); Alginate oligosaccharides (AOSs); Middle-viscosity sodium alginate (MV-Algin); Low-viscosity sodium alginate (LV-Algin); Guluronate oligosaccharides (polyG); Mannuronic oligosaccharides (polyM); Polysaccharide lyase (PL); Carbohydrate-active enzymes (CAZy); Guluronic acid monosaccharide (G1); Guluronic acid disaccharide (G2); Guluronic acid trisaccharide (G3); Guluronic acid tetrasaccharide (G4); Guluronic acid pentasaccharide (G5); Guluronic acid hexasaccharide (G6); Guluronic acid heptasaccharide (G7); Mannuronic acid monosaccharide (M1); Mannuronic acid disaccharide (M2); Mannuronic acid trisaccharide (M3); Mannuronic acid tetrasaccharide (M4); Mannuronic acid pentasaccharide (M5); Mannuronic acid hexasaccharide (M6); Mannuronic acid heptasaccharide (M7); Unsaturated guluronic acid disaccharide (△G2); Unsaturated guluronic acid trisaccharide (△G3); Unsaturated guluronic acid tetrasaccharide (△G4); Unsaturated guluronic acid pentasaccharide (△G5); Unsaturated guluronic acid hexose (△G6); Yeast extract peptone dextrose (YPD); Buffered minimal methanol medium (BMMY); Dissolved oxygen (DO); Liquid chromatography-electrospray ionization-MS (LC-ESI-MS); Polymerization (DP); Root-mean-square deviation (RMSD); Scanning electron microscopy (SEM); 3,5-dinitrosalicylic acid (DNS).
