# Supplementary material for: Degradation of Natural Undaria pinnatifida into Unsaturated Guluronic Acid Oligosaccharides by a Single Alginate Lyase
Source: Mar Drugs. 2024 Oct 2;22(10):453. doi: 10.3390/md22100453 (PMC11509462; doi:10.3390/md22100453)
Supplement: Supplementary file 1 [file marinedrugs-22-00453-s001.zip › marinedrugs-3201355-supplementary.pdf]

Supplementary Information for:

Degradation of Natural *Undaria pinnatifida* into Unsaturated Guluronic Acid Oligosaccharides by a Single Alginate Lyase

Authors: Hui Wang [a], Jiaqi Wen [a], Nuraliya Ablimit [a], Kun Deng[a], Wenzhuo Wang [a], Wei Jiang [a,*]

Affiliations: [a] State Key Laboratory of Animal Biotech Breeding, College of Biological Sciences, China Agricultural University, Beijing 100193, China.

***Corresponding author:** Prof. Wei Jiang. **E-mail:** jiangwei01@cau.edu.cn. **Tel:** 8610-62731440.

E-mail addresses of other authors:

Hui Wang: wh15666529202@163.com

Jiaqi Wen: wjq18810755707@163.com

Nuraliya Ablimit: aliya0814@163.com

Kun Deng: dengkun1103@163.com

Wenzhuo Wang: sz20213020199@cau.edu.cn

Present address: College of Biological Sciences, China Agricultural University, Yuan Ming Yuan West Road No. 2, Haidian District, Beijing 100193, China.

Contents

Fig. S1. PCR confirmation of constructed engineered strains.

Fig. S2. Agitation and aeration variation in high-density fermentation of *p-Alg* in the 7.5-L fermenter.

Fig. S3. Enzymatic characteristics of Vnalg7 in engineered strain p-ALG.

Fig. S4. Multiple-sequence alignment of Vnalg7 and PL7 family ALGs cited in this study.

Fig. S5. LC-MS analysis of LV-Algin and guluronate oligosaccharide standards with various DP values.

Fig. S6. HPLC analysis of Vnalg7-catalyzed hydrolysates of mannuronate oligosaccharides.

Fig. S7. Predictive 3D model of Vnalg7 by AlphaFold2 (purple) and SWISS-MODEL (cyan).

Fig. S8. SDS-PAGE analysis of Vnalg7 and its mutants.

Fig. S9. Enzymatic activity of Vnalg7 and its mutants.

Fig. S10. Schematic representation of Vnalg7 active pocket positions and intercatalytic site distances.

Table S1. Oligonucleotide primers used in this study.

Table S2. Effects of metal ions (5 mM solution) and chemical reagents on Vnalg7 activity.

Table S3. Kinetic parameters K_m and V_{max} of Vnalg7 toward various substrates.

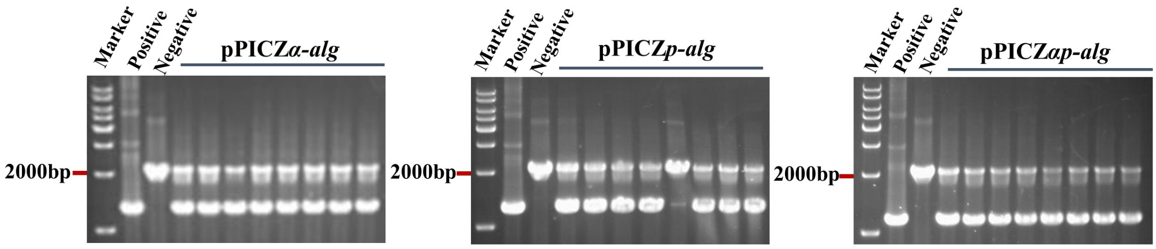

Fig. S1. **PCR confirmation of constructed engineered strains.** Positive variants and *P. pastoris* X-33 (control strain, without target gene) were identified using the PCR primer pair AOX-F/AOX-R. Marker: 1-kb DNA markers. Positive: plasmids pPICZα-*alg*, pPICZp-*alg*, and pPICZαp-*alg*. Negative: X-33.

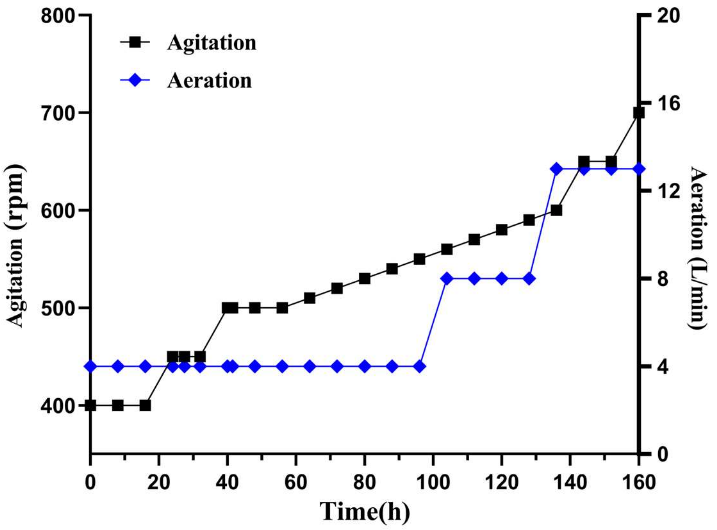

Fig. S2. **Agitation and aeration variation in high-density fermentation of *p-Alg* in the 7.5-L fermenter.** Black square, agitation. Purple circle, aeration.

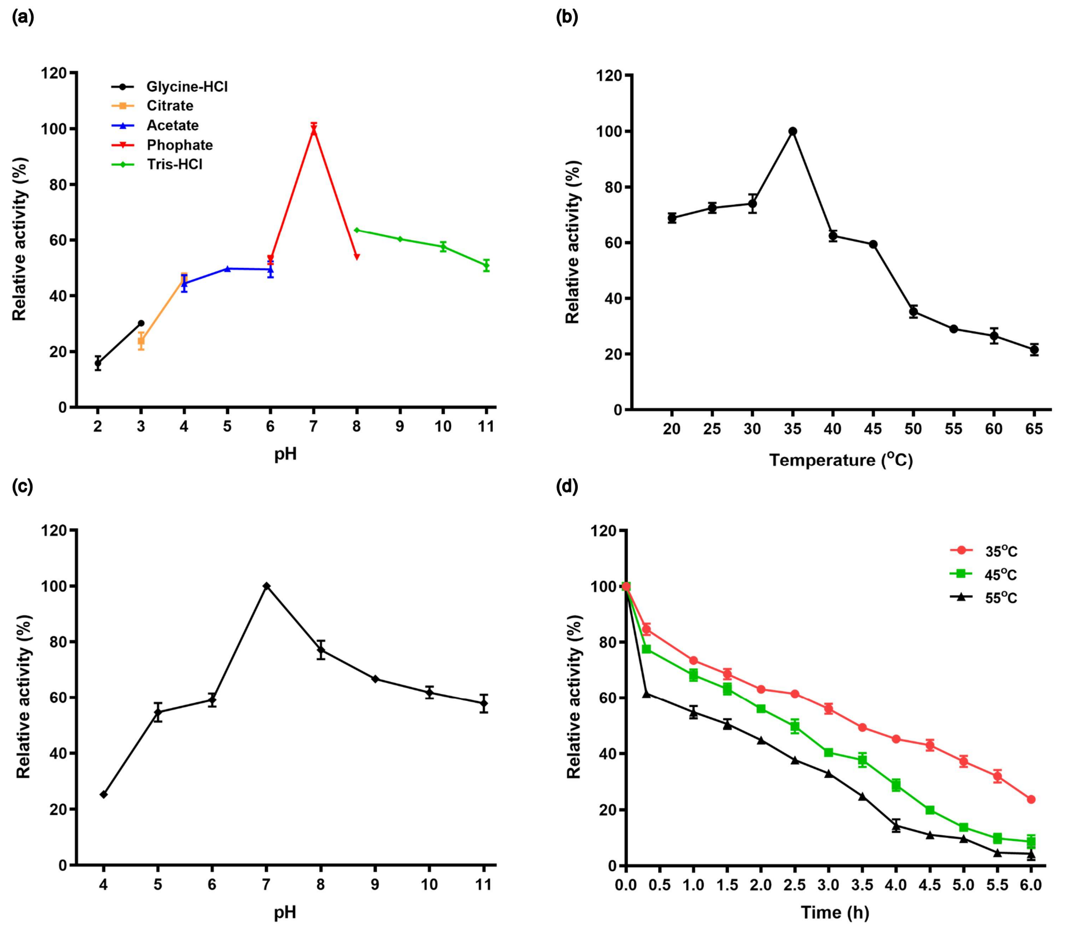

Fig. S3. **Enzymatic characteristics of Vnalg7 in engineered strain *p-Alg*.** **(a)** Effects of pH on enzyme activity. **(b)** Effects of temperature on enzyme activity. **(c)** pH stability following 1-h preincubation in pH buffers shown in (a). **(d)** Thermostability in phosphate buffer, at three temperatures.

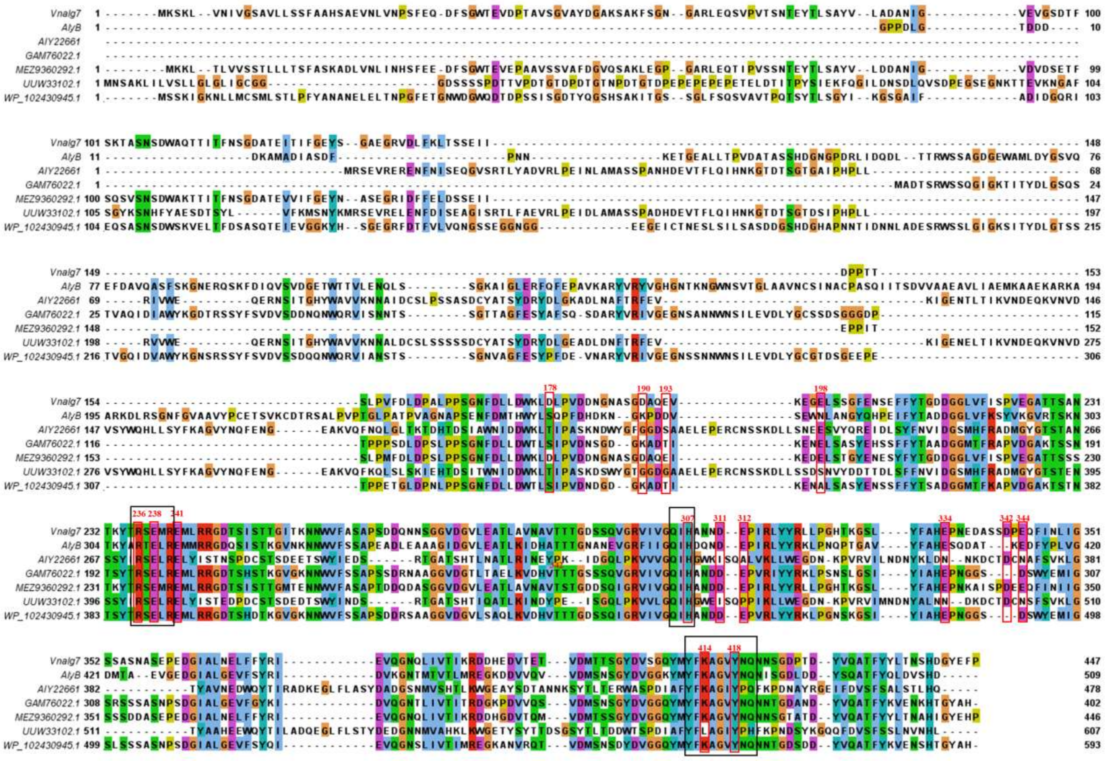

Fig. S4. Multiple-sequence alignment of Vnalg7 and PL7 family ALGs cited in this study. Residues Glu^{193}, Glu^{198}, Glu^{238}, Glu^{241}, Glu^{312}, Glu^{334}, Glu^{344}, Asp^{178}, Asp^{190}, Asp^{311}, Asp^{342}, Arg^{236}, His^{307}, Lys^{414}, and Tyr^{418} (order number in Vnalg7) which may participate in the catalytic process are highlighted in red. Three conserved regions in the PL7 family: QI(V)H, RXEL(V)R, and YFKXGXYXQ are boxed in black.

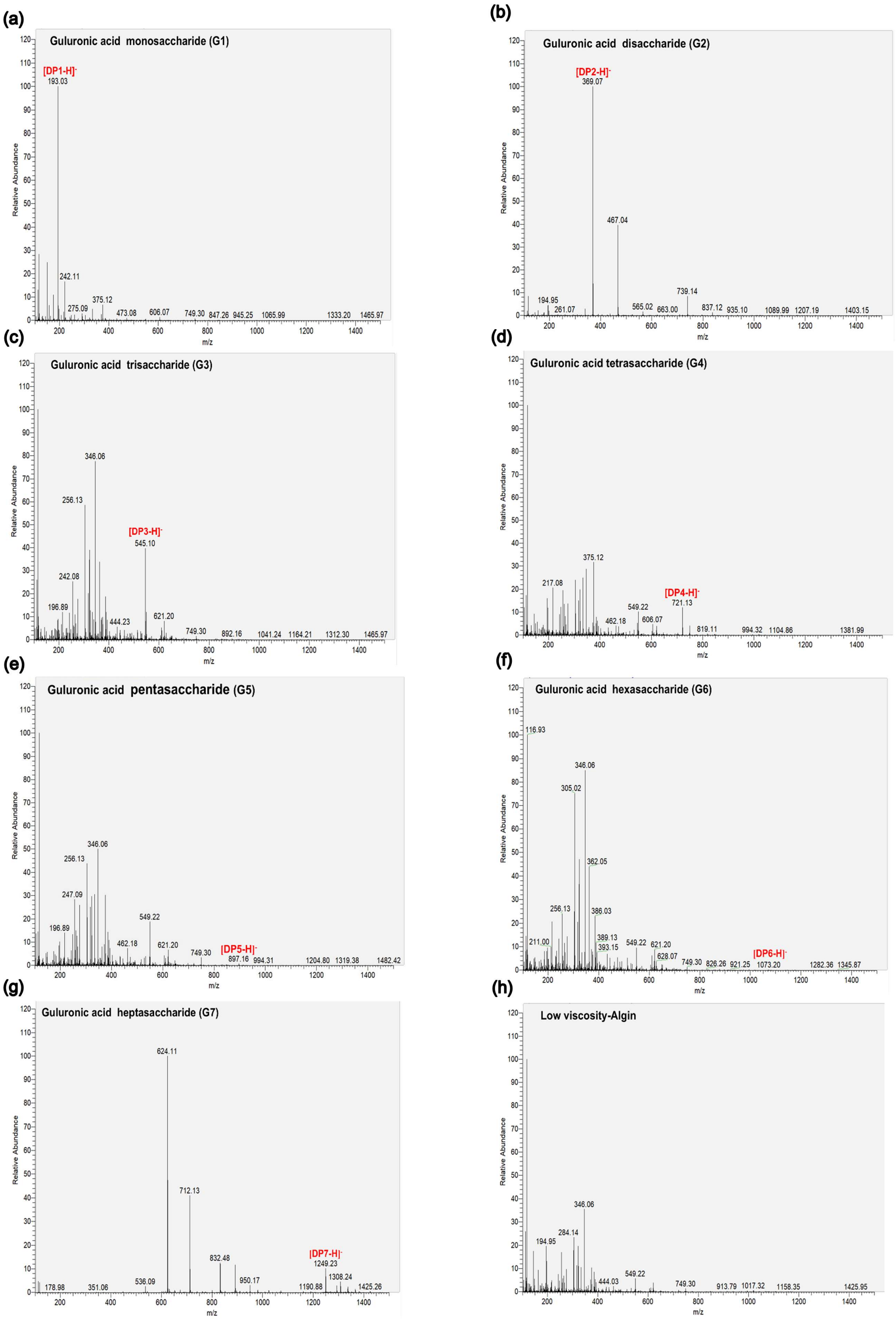

Fig. S5. LC-MS analysis of LV-Algin and guluronate oligosaccharide standards with various DP values.

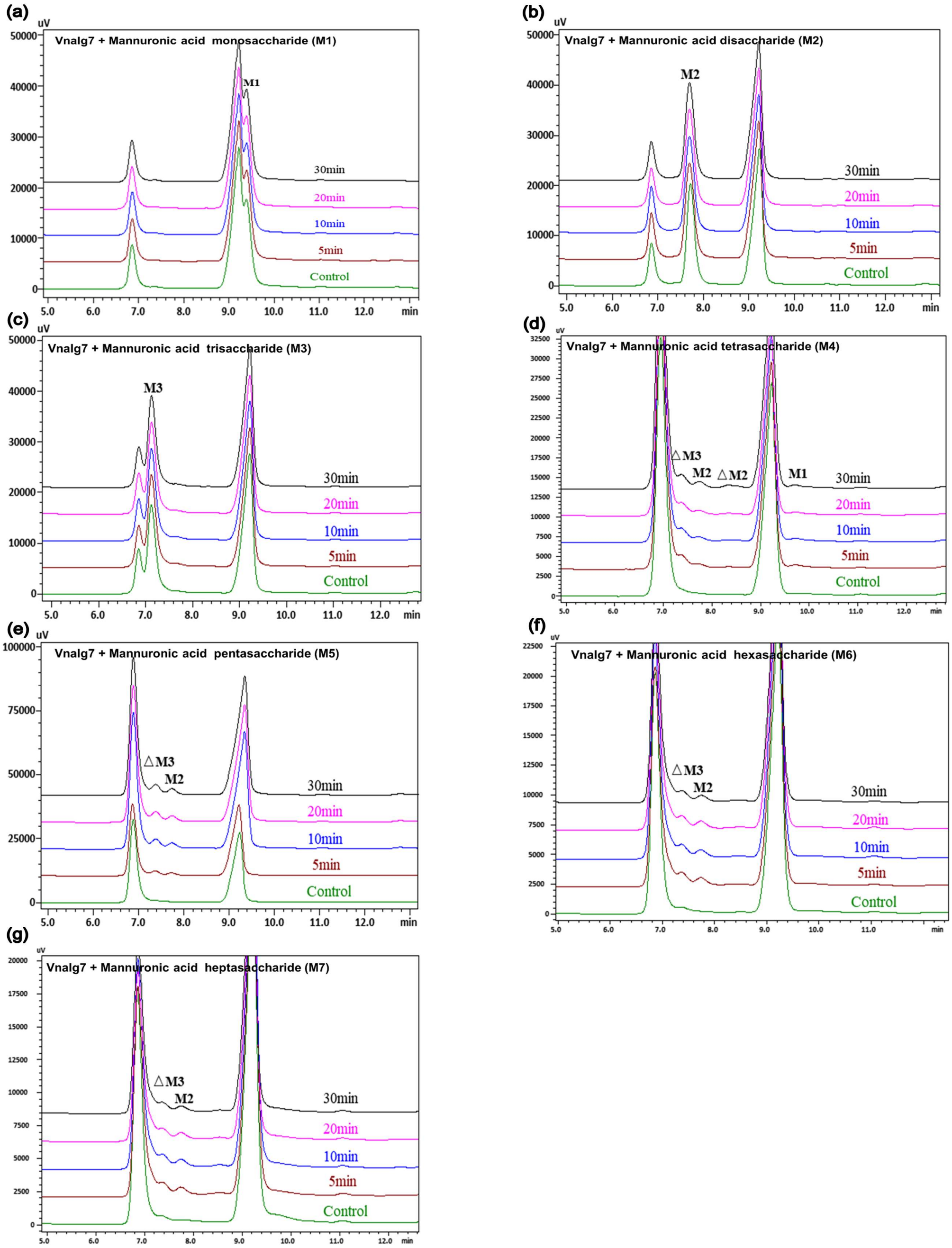

Fig. S6. HPLC analysis of Vnalg7-catalyzed hydrolysates of mannuronate oligosaccharides. Time course of Vnalg7-catalyzed hydrolysis of mannuronic acid oligosaccharides with various DP values. **(a-g)** mannuronic acid monosaccharide (M1), disaccharide (M2), trisaccharide (M3), tetrasaccharide (M4), pentasaccharide (M5), hexasaccharide (M6), and heptasaccharide (M7), respectively, as indicated in panel captions. Control: reaction system with inactivated Vnalg7 enzyme solution.

Fig. S7. Predictive 3D model of Vnalg7 by AlphaFold2 (purple) and SWISS-MODEL (cyan).

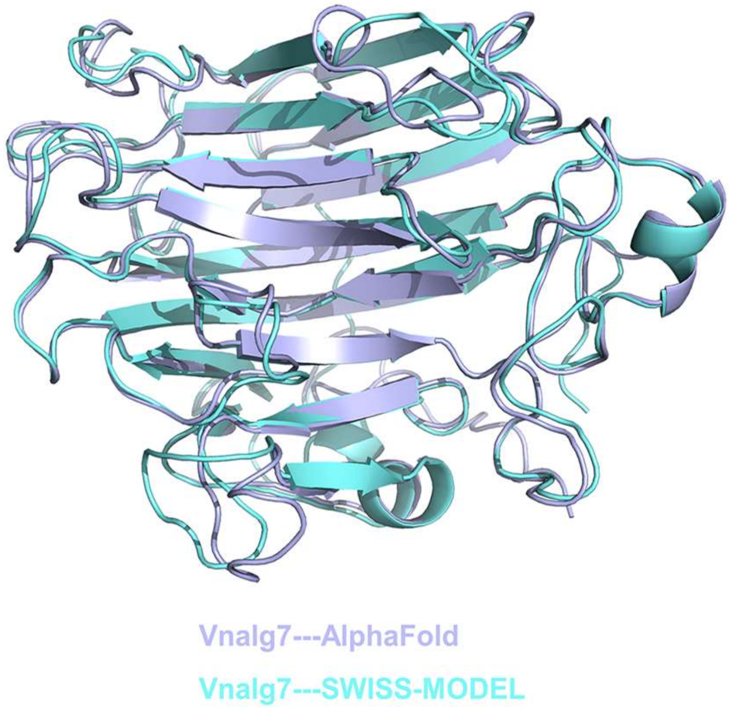

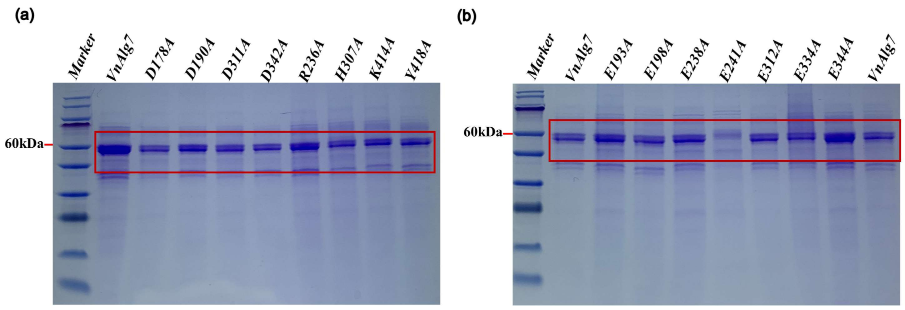

Fig. S8. **SDS-PAGE analysis of Vnalg7 and its mutants.** Marker: PM2510 standard protein Mw markers.

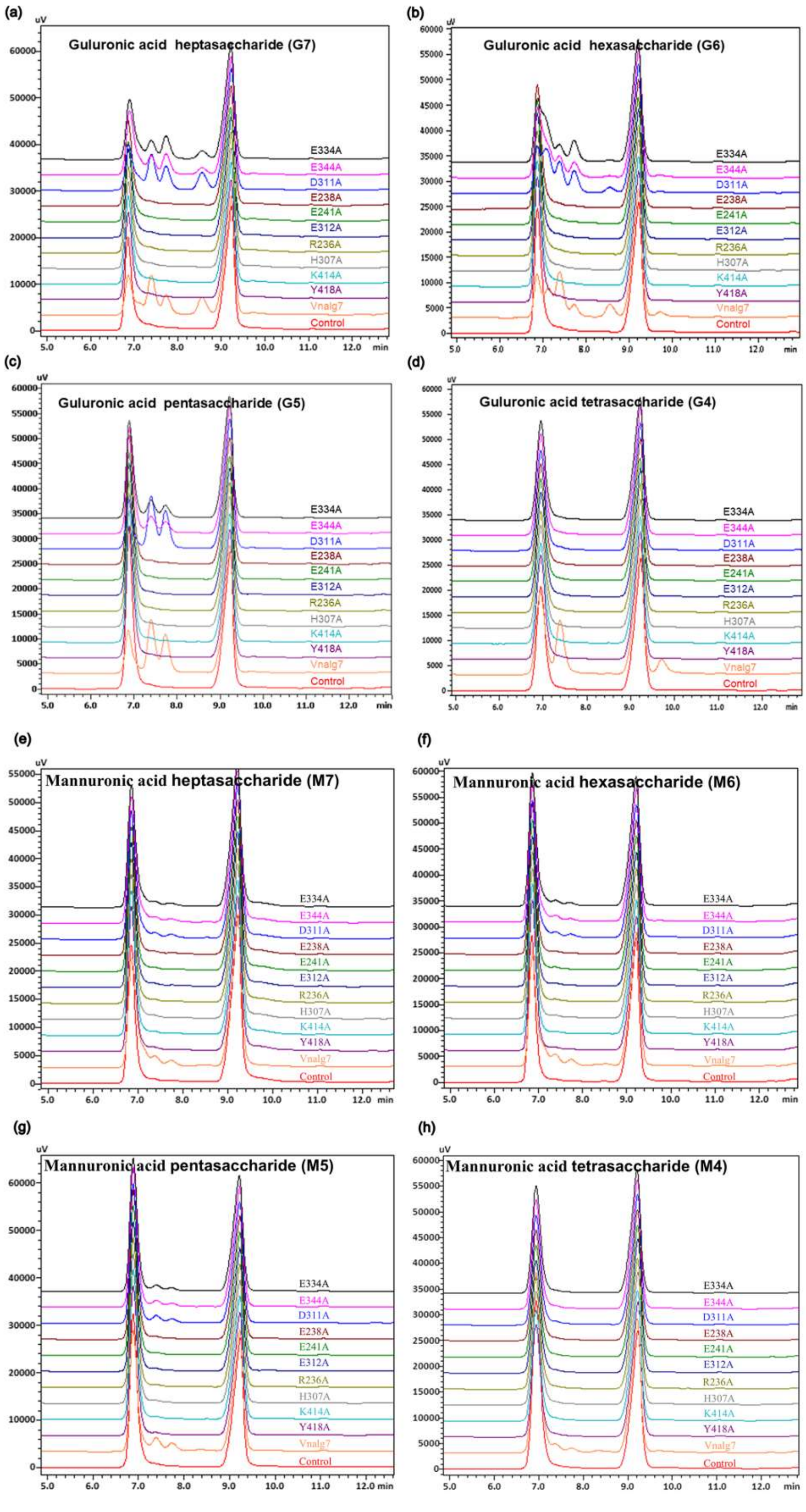

Fig. S9. Enzymatic activity of Vnalg7 and its mutants. HPLC analysis of hydrolysates of substrates G7 **(a)**, G6 **(b)**, G5 **(c)**, and G4 **(d)**. **(e-h)** Hydrolysates of M7, M6, M5, and M4, respectively, as indicated in panel captions. Control as in Fig. S6.

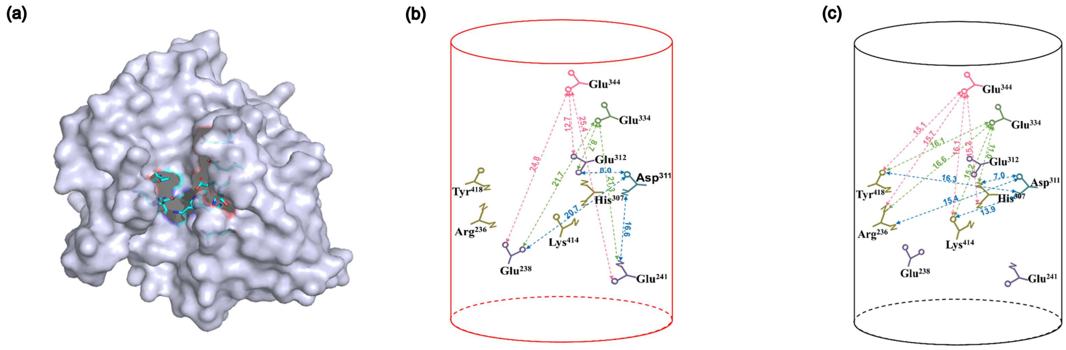

Fig. S10. Schematic representation of Vnalg7 active pocket positions and intercatalytic site distances. (a) Surface representation of 3D structure of Vnalg7, showing catalytic amino acid residues in predicted active pocket. (b, c) Schematic structural representations of catalytic region, with distances (Å) between catalytic residues indicated.

Table S1. Oligonucleotide primers used in this study.

primer	sequence (5´- 3´)
pPICZα-*alg*-F	GAAAAGAGAGGCTGAAGCTGAATTCGCCGAAGTTAACTTGGTTAACCCA
pPICZα-*alg*-R	TGGGTTAACCAAGTTAACTTCGGCGAATTCAGCTTCAGCCTCTCTTTTC
pPICZp-*alg*-F	AAAAAACAACTAATTATTCGAAACGGAATTCATGAAGTCCAAGTTGGTT
pPICZp-*alg*-R	AACCAACTTGGACTTCATGAATTCCGTTTCGAATAATTAGTTGTTTTTT
AOX-F	GACTGGTTCCAATTGACAAGC
AOX-R	GCAAATGGCATTCTGACATCC
D178-F	TGGAAGTTGGCTTTGCCAGTTGACGATAACGGTAACGCTTCTGGTG
D178-R	AACTGGCAAAGCCAACTTCCAATCCAAAAGATCAAAGTTACCAGAA
D190-F	GCTTCTGGTGCTGCTCAAGAAGTTAAGGAGGGTGAATTGTCTTCTG
D190-R	TTCTTGAGCAGCACCAGAAGCGTTACCGTTATCGTCAACTGGCAAA
E193-F	GATGCTCAAGCTGTTAAGGAGGGTGAATTGTCTTCTGGTTTTGAAA
E193-R	CTCCTTAACAGCTTGAGCATCACCAGAAGCGTTACCGTTATCGTCA
E198-F	AAGGAGGGTGCTTTGTCTTCTGGTTTTGAAAATAGTGAATTTTTCT
E198-R	AGAAGACAAAGCACCCTCCTTAACTTCTTGAGCATCACCAGAAGCG
R236-F	TACACTGCTAGTGAAATGAGAGAGATGTTGAGAAGAGGTG
R236-R	TTCACTAGCAGTGTACTTAGTGTTAGCAGAAGTAGTAGCTCCT
E238-F	ACTAGAAGTGCTATGAGAGAGATGTTGAGAAGAGGTGATACTTCTA
E238-R	CTCTCTCATAGCACTTCTAGTGTACTTAGTGTTAGCAGAAGTAGTAGCT
E241-F	ATGAGAGCTATGTTGAGAAGAGGTGATACTTCTATTAGTACTA
E241-R	CAACATAGCTCTCATTTCACTTCTAGTGTACTTAGTGTTAGCA
H307-F	GGTCAAATTGCTGCTAACAACGATGAACCAATTAGATTGTACTATA
H307-R	GTTGTTAGCAGCAATTTGACCAACAATAACTCTACCGACTTGAGAA
E312-F	AACGATGCTCCAATTAGATTGTACTATAGATTGTTGCCAG
E312-R	AATTGGAGCATCGTTGTTAGCATGAATTTGACCAACAATA
D311-F	GCTAACAACGCTGAACCAATTAGATTGTACTATAGATTGTTGCCAG
D311-R	AATTGGTTCAGCGTTGTTAGCATGAATTTGACCAACAATAACTCTA
E334-F	TTTGCTCATGCTCCTAACGAAGATGCCAGTTCCGATCCAGAACAAT
E334-R	TTCGTTAGGAGCATGAGCAAAGTACAAACTACCTTTAGTATGACCT
D342-F	GCCAGTTCCGCTCCAGAACAATTTATTAACTTGATTGGATCTTCCG
D342-R	TTGTTCTGGAGCGGAACTGGCATCTTCGTTAGGTTCATGAGCAAAG
E344-F	GATCCAGCTCAATTTATTAACTTGATTGGATCTTCCGCCT
E344-R	AAATTGAGCTGGATCGGAACTGGCATCTTCGTTAGGTTCA
K414-F	ATGTACTTTGCTGCTGGTGTTTACAATCAAAACAATTCAGGTGATC
K414-R	AACACCAGCAGCAAAGTACATATACTGACCAGAGACATCATAACCT
Y418-F	GCTGGTGTTGCTAATCAAAACAATTCAGGTGATCCAACTGATTACG
Y418-R	GTTTTGATTAGCAACACCAGCCTTAAAGTACATATACTGACCAGAG

Table S2. Effects of metal ions (5 mM solution) and chemical reagents on Vnalg7 activity.

ion or reagent	relative activity (%)	ion or reagent	relative activity (%)
control	100.00 ± 0.70	Ca^{2+}	73.83 ± 0.89
Ni^{2+}	44.87 ± 5.89	Cu^{2+}	26.29 ± 5.51
Co^{2+}	51.57 ± 2.41	Mg^{2+}	96.21 ± 1.72
Al^{3+}	67.88 ± 1.46	K^{+}	92.18 ± 2.94
Na^{+}	100.13 ± 3.43	SDS (0.1%)	21.36 ± 3.14
Mn^{2+}	71.68 ± 1.09	EDTA	78.88 ± 2.11
Zn^{2+}	46.64 ± 3.87	Tween-20 (0.05%)	113.66 ± 3.95

Table S3. Kinetic parameters K_m and V_{max} of Vnalg7 toward various substrates.

substrate	K_m (mg/mL)	V_{max} (U/mg)
middle-viscosity sodium alginate (MV-Algin)	4.60 ± 1.10	2000.00 ± 13.50
low-viscosity sodium alginate (LV-Algin)	5.00 ± 0.34	1666.67 ± 14.96
poly-α-L-guluronate (polyG)	5.47 ± 0.14	1833.34 ± 15.57
poly-β-D-mannuronate (polyM)	7.42 ± 0.09	1166.67 ± 14.12

The enzyme protein concentration used in the kinetic parameter determination experiments was 1.32mg/mL, and 0.10 mL was added.
